# Viral load testing and the use of test results for clinical decision making for HIV treatment in Cameroon: An insight into the clinic-laboratory interface

**DOI:** 10.1371/journal.pone.0198686

**Published:** 2018-06-11

**Authors:** George Awungafac, Elvis T. Amin, Akemfua Fualefac, Noah F. Takah, Lucy A. Agyingi, Julius Nwobegahay, Pascale Ondoa, Patrick A. Njukeng

**Affiliations:** 1 African Society for Laboratory Medicine, Addis Ababa, Ethiopia; 2 Ministry of Public Health, Yaounde, Cameroon; 3 Global Research Education and Health Foundation, Buea, Cameroon; 4 Global Health Systems Solutions, Limbe, Cameroon; 5 International Diagnostic Centre (IDC), London School of Hygiene and Tropical Medicine (LSHTM), London, United Kingdom; 6 Faculty of Science, University of Dschang, Dschang, Cameroon; 7 Military Health Research Center (CRESAR), Yaounde, Cameroon; University of KwaZulu-Natal, SOUTH AFRICA

## Abstract

**Background:**

The viral load (VL) in patients receiving antiretroviral therapy (ART) is the best predictor of treatment outcome. The anticipated benefits of VL monitoring depend on the actual uptake of VL test results for clinical decisions. The objective of this study was to assess the uptake and utilization of VL test results for clinical decisions on HIV treatment in Cameroon, from 2013 to 2017.

**Methods:**

This was a retrospective cohort analysis of data from files of patients receiving ART at Buea, Limbe, Bamenda and Bafoussam regional hospital HIV treatment centers. A simple random pick of six file blocks was performed in each shelf that corresponded to a year of initiation, and the contents of all selected files were reviewed and the information needed for the study entered a structured questionnaire. The data collected was recorded in Epi Info (version 7.1.5.2), and analyzed using SATA (version 12.1; StataCorp LP).

**Results:**

Eight hundred and thirty files were reviewed. The mean duration on ART was 39.4±12 months. Viral load testing uptake was 24.33% and only one VL test had been done by all patients. Approximately 65% of the patients did the first VL after more than 24 months on ART. The median turnaround (TAT) time for VL testing was 6 days (Interquartile range (IQR) 3-7days). Among 201 patients who did a VL test, 94.55% had VL suppression (≤1000copies/mm^3^). Approximately 54% of the patients with virologic failure were switched to a second-line regimen.

**Conclusions:**

The uptake of viral load testing is low in North West, South West and West Regions of Cameroon. The current TAT for VL testing is plausible. The rate of switch to second line regimen is low. It is time to strengthen the scale up of VL testing and improve the rate of switch to second-line regimen in Cameroon.

## Background

In December 2013, the Joint United Nation’s Program on HIV/AIDS put into action a ‘90-90-90’ strategy with the goal of bringing an end to the AIDS epidemic [1]. The third ‘90’ in the strategy will ensure that all people receiving antiretroviral therapy (ART) are virologically suppressed by 2020. This strategy requires countries to roll out, decentralize and sustain routine viral load (VL) monitoring of all people on ART.

In Cameroon, where the HIV epidemic remains generalized, with an estimated prevalence of 4.8% in 2014, and 660,000 people living with HIV (PLWH) [2], immunological testing of CD4 cell count has been the benchmark for ART monitoring. Before now, the situation of ART monitoring in Cameroon and several Sub-Saharan African countries contrasted with what obtains in HIV programs of developed countries where VL testing is routine [3,4]. Antiretroviral treatment outcome has been shown to be best predicted by consecutive measurements of the viral load (VL) [5]. Within a programmatic set up, virologic data provides useful information about the impact of ART: At the patient level, sustained VL suppression is shown to prevent the emergence of HIV drug resistance (HIVDR) [6–8]; decreased VL is associated with a reduction in HIV incidence at the population level [9,10] and VL data helps in patient monitoring [11–13].

In 2014, Cameroon adopted the 2013 World Health Organization’s recommendation of VL testing as a preferred approach for diagnosis and confirmation of ART failure [14,15]. However, until 2017, routine VL assessment of ART patients has not been part of a normal practice in the country [16]. A targeted VL testing strategy, however, has been implemented among treatment cohorts in specialized HIV treatment units in some regions including the North West, South West and the West where this study was conducted. Targeted VL testing can be offered to HIV ART patients who show persistently poor clinical and or immunological responses to ART despite evidence of good adherence [17]. Cross-sectional assessments conducted at the sites in North West and South West Regions have reported virologic failure (VF) (viral load >1000copies/ml) of 23.2% at 16 months [18], and 17.6% at 36 months [19] in clinics from Yaoundé.

New international guidelines recommend laboratory assessments of VL at 6 months, 12 months and thereafter every 12 months after beginning ART in settings where VL testing is available, and a discontinuation of CD4 cell count monitoring in ART stable patients [20]. These consolidated guidelines of the WHO have been adopted by the Cameroon ministry of public health (MOPH), and is beginning to prepare for the scale-up of routine VL monitoring [15]. A VL of more than 1000 HIV RNA copies/mL is an indication to intensify patient adherence counselling or to switch to more effective regimens. Studies have shown that the mortality rate of patients with VF who are switched to a second-line regimen is significantly lower than those not switched or when the switch is delayed [21]. In the face of the new guidelines, the anticipated benefits of VL monitoring will largely depend on the actual use of VL test results for clinical decision making and to successfully address the treatment options and needs of HIV infected individuals.

The availability of equipment, challenges in transportation of samples and test results, requisition of VL tests by clinicians and the readiness for a clinical decision upon results reception are potential barriers that can hinder the effective scale up of VL and render VL less economically beneficial. Data on possible barriers and challenges to the optimal uptake of VL testing and use of test results are needed to inform the operationalization of routine VL testing at the national scale. The availability of data on VL uptake in this era of expansion of VL testing will strengthen understanding on the clinic-laboratory interactions and serve as potential advocacy tools that could lead to improvement of the monitoring of ART.

The focus of this study was to assess the level of and determinants limiting the uptake and utilization of VL test results for clinical decisions on HIV treatment in Cameroon, from 2013 to 2017. The objectives were to i) assess coverage of routine VL testing among patients receiving ART in 3 regions of Cameroon ii) determine the turnaround time for VL testing iii) determine the rate of virologic suppression using the threshold of 1000copies /mL and iv) assess clinicians’ decisions following a VL test result.

## Methods

### Study design and setting

The study was a retrospective cohort analysis of data from files of patients receiving ART at regional HIV referral treatment centers. We purposively selected 3 neighboring regions of Cameroon with a comparatively high HIV burden and ART enrolment rate; South West, North West and West Regions for participation. According to a national report of 2015, the North West, South West and the West regions represented respectively 15%, 11% and 9% of the HIV patients on ART in Cameroon [22]. In the participating regions, targeted VL was usually conducted at the regional hospitals. In South West Region, data was collected at Limbe and Buea Regional Hospital treatment centres; in the North West at the Bamenda Regional Hospital and Bafoussam Regional Hospital in the West Region. Except for Bamenda and Bafoussam, dried blood spot (DBS) for viral load testing are normally collected at the treatment centers and ferried to central labs in Yaounde for continuation of the viral load testing process. At the time of the study, plasma samples were used for onsite VL testing in the Bamenda Regional Hospital while DBS specimen was collected from patients in Bafoussam Regional Hospital and transported to nearby Dschang for VL testing. Viral load test results followed a similar route for return to the facility.

### Ethics statement

Ethical clearance was obtained from the National Ethics Committee of Research for Human Health (NECRHH) (No.2017/04/899). We ensured full compliance to the terms for implementation of the protocol as prescribed by the Institutional Review Board (IRB), and abided to the administrative procedures laid down by the Ministry of Health, Cameroon. Even though the NECRHH accepted a waiver for informed consent, we also applied and obtained a waiver of informed consent from the directors of the hospitals involved. No names or other information that could personally identify the patient was collected. We used a coding system whereby we established a combination of figures each patient record, to link clinic identification codes used at the treatment center to the codes assigned for each participant in the study.

### Inclusion and exclusion criteria

Eligible files were those of people living with HIV (PLWH) who have been receiving ART for a minimum of 1.5 (i. e 18 months), aged >18 years and alive at the time of data abstraction. A 1.5 years limit was chosen because it gives us more information about VL testing recommendations which require that by 1.5 years, patients on ART should have received at least 2 VL assessments. During a preliminary assessment of the filing system at the treatment centres, we observed that there were more adults’ files. To avoid under-representation, we therefore did not consider participants less than 18 years. Files of patients transferred in or transferred out, including those of patients who were loss to follow up for more than a year were excluded.

### Sampling and data collection

At the files rooms, the room keeper showed the data collector the order of arrangement of the files. In all the treatment centers this was organized by year, and within each year there was monthly separation of files based on the date of start of ART. In each of the yearly shelf, we did a simple random pick of six file blocks, each of which corresponded to files of all patients initiated into ART during the month. The sampling frame prepared in advance comprised of all monthly file blocks for a year of initiation for each of 2013, 2014 and 2015. For each year, the months were each given numeric codes. We then wrote down the code of each month on a tiny piece of paper and concealed it for six random picks from the twelve concealed codes. The file blocks of the month corresponding to the selected code were considered. A training on the sampling frame and the sampling and data collection was provided to 3 data collectors. All the files in each selected file block for the cohorts (patients ART-initiated) of 2013, 2014 and 2015 were reviewed. A structured questionnaire was developed on Epi Info version 7.0 to capture data on socio-demographic characteristics, treatment initiation, viral load testing and change of treatment regimen.

### Data management and analysis

#### Analysis plan

Data was analyzed using STATA (version 12.1; StataCorp LP, College Station, TX). In preparing our dataset for analysis, the original set, a Microsoft Excel sheet (exported from Epi Info), was converted into the necessary STATA (.dta) formats. Descriptive statistics was conducted for the socio-demographic characteristics, to assess uptake of viral load testing, frequency of treatment change and criteria for change of regimen. The output of descriptive statistics was presented using tables. Key definitions used during analysis are described in Table 1.

**Table 1:** Definition of key analysis terms and measures.

Term	Definition	Variable measure
Overall Uptake (or coverage) of viral load testing	This is the proportion ART patients for whom a viral load was requested and the results received at the facility at.	**Numerator**: Total number of patients for whom a VL test result was received at facility: **Denominator**: Total number of patients who should have received a VL test
Uptake (or coverage) of timely viral load testing	We defined uptake regarding the timing of VL testing among the patients. We assessed the proportion of ART patients with a VL test that was done as per recommendations: 0-6months, 7-12month, 13–24 months and more than 24 months on ART.	**Numerator**: number of patients for whom a VL test result was received at facility at the time on ART: **Denominator**: Total number of patients who needed a VL test at the time on ART
Use of viral load test results	This refers to decision making by clinicians following a VL test result. It is either a switch or a substitution or no change in regimen. A substitution is a manipulation of the first-line regimen while a switch is a change to second-line treatment. Lack of regimen change means two-fold; i) a conscious clinical decision to continue with same regimen (VL suppression); ii) not acting to switch to second-line (VF). Poor adherence information has potential to limit the ability to take a clinical decision despite evidence of viral replication	Switch or substitution, no regimen change
Turnaround time (TAT) for viral load testing	The duration in days from date of VL test request to the date the results were received in the facility. This may not be very precise because the date of test request may not actually be the date the blood sample was collected. We used the date the test was requested as a proxy for the date sample was collected. We did not study how different phase of the lab testing cycle affected turnaround time	Date of request of VL tests, date of receipt of VL result, **date difference (days**)
Clinical outcomes	This refers to whether there was a viral load suppression or virologic failure. It was assessed based on the VL test results available. We did not assess quality of life and mortality as outcomes in this study. We caution readers to the fact that in the event where the current WHO VL testing guidelines were not respected, virologic failure or suppression cannot be ascertained based on only one VL test result.	Suppressed (undetectable viral load, i. e ≤1000RNA copies/ml), failure (>1000 RNA copies/ml detected)

## Results

### Presentation of participants

Eight hundred and thirty files (830) were reviewed; 24.2%, 15.4%, 24.5% and 35.8% from Bamenda, Bafoussam, Buea and Limbe Regional Hospitals respectively. The median age of study participants was 40 years (IQR: 34.5–47.5 years). Approximately 65% of the study population were females and 55% were married. The median delay to begin ART following HIV diagnosis was 1 month (IQR: 1–5.5 months). More participants were enrolled into ART at WHO clinical stage 3 (36%) of HIV infection. The mean duration on ART was 39.4±12.90 months (Table 2).

**Table 2:** Clinical characteristics of HIV patients who initiated antiretroviral therapy from January 2013-December 2015 in four regional hospitals in Cameroon (n = 830).

Characteristic	Frequency (N, %)		
	Total	Male (n = 290)	Female (n = 540)
**Year of initiation**			
2013	326 (39.61)	107 (37.02)	219 (41.01)
2014	229 (27.83)	83 (28.72)	146 (27.34)
2015	268 (32.56)	99 (34.26)	169 (31.65)
**Age group**			
19–28	89 (10.72)	10 (3.45)	79 (14.63)
29–38	272 (32.77)	76 (26.21)	196 (36.30)
39–48	290 (34.94)	126 (43.45)	164 (30.37)
49–58	133 (16.02)	61 (21.03)	72 (13.33)
59–68	36 (4.34)	12 (4.14)	24 (4.44)
69–78	10 (1.20)	5 (1.72)	5 (0.93)
**Marital status (n = 830)**			
Single	299 (36.07)	82 (28.28)	217 (40.85)
Married	456 (55.01)	180 (62.07)	276 (51.11)
Divorced	40 (4.83)	11 (3.79)	29 (5.37)
Cohabiting	34 (4.10)	16 (5.52)	18 (3.33)
**Education (n = 814)**			
None	68 (8.19)	15 (5.17)	53 (9.81)
Primary	357 (43.01)	128 (44.14)	229 (42.41)
Secondary	341 (41.08)	124 (42.76)	217 (40.85)
Tertiary	64 (7.71)	23 (7.93)	41 (7.59)
**Smoking history (n = 824)**			
No	780 (93.98)	248 (85.52)	532 (98.52)
Yes	44 (5.30)	40 (13.79)	4 (0.74)
**Alcohol history (n = 827)**			
none	451 (54.53)	141 (48.62)	310 (57.41)
Mild	283 (34.22)	94 (32.41)	189 (35.00)
Moderate	65 (7.86)	33 (11.38)	32 (5.93)
Heavy	28 (3.39)	21 (7.24)	7 (1.30)
**Household size**	4.21 ± 2.53)	4.41±2.83	4.17±2.36
**Clinical stage, WHO (n = 824)**			
I	230 (27.91)	72 (24.83)	158 (29.26)
II	212 (25.73)	81 (27.93)	131 (24.26)
III	298 (36.17)	101 (34.83)	197 (36.48)
IV	84 (10.19)	36 (12.41)	48 (8.89)
**Baseline weight (Mean±SD)**	65.97± 13.68	67.65±11.85	65.09±14.50
**Months since diagnosis to start of ART (Median, IQR)**	1: <1–5.5	1: 0–6	1: 0.5–5.5
**Months on ART (Mean)**			
All patients	39.4±12.9	38.7±11.3	39.7±13.6
Virologic failure	39.4±12.1	35.5±13.6	44.0±9.4
Viral load suppression	36.0±14.0	34.5±10.2	36.9±15.7

### The uptake of viral load testing

This is demonstrated in Table 3. Six patients (0.73%) had a viral load (VL) test done before starting ART. The uptake of VL testing after starting ART was 24.33% among the study population. No participant did more than one VL test during the time on ART. A VL test was more likely to be done by patients who have stayed longer on treatment [p(^x2^ trend) <0.0001]. The uptake of VL testing significantly improved with time [p(x^2^ trend) = 0.012]. The median turnaround (TAT) time for VL testing was 6 days (IQR: 3-7days). Viral load suppression was observed in 94.53% of patients who did a VL after starting ART.

**Table 3:** Profile of uptake of laboratory monitoring of viral load testing among HIV patients in 2017 who initiated ART in 2013, 2014 and 2015 in four regions hospitals of Cameroon (n = 830).

Variable	Freq (N, %)	Freq. Female (N, %)	Freq. Male (N, %)	p- value (Chi Trend on Total)
**In ART**	201 (24.33)	124 (22.96)	77 (26.55)	
**Year of ART initiation**				
2013 (n = 326)	59 (18.10)	40 (18.26)	19 (17.76)	0.012
2014 (n = 229)	58 (25.33)	31 (21.23)	27 (32.53)	
2015 (n = 268)	79 (29.48)	48 (28.40)	31 (31.31)	
**Timing of first VL during ART (n = 201)**				
Up to 6 months	3 (1.49)	2 (1.61)	1 (1.31)	<0.0001ⱡ
7–12 months	14 (6.97)	9 (7.26)	5(6.49)	
13–24 months	52 (25.87)	31(25.00)	21 (27.27)	
>24 months	132 (65.67)	82(66.13)	50(64.94)	
**Pre-CD4 count**				
CD4<500 (n = 579)	140 (24.18)	80 (22.29)	60 (27.40)	
CD4>500 (n = 239)	58 (24.27)	43 (24.43)	15 (23.81)	
**Marital status**				
Single (n = 296)	45 (15.20)	32 (14.95)	13 (16.25)	
Married (n = 455)	132 (29.01)	80 (29.09)	52 (28.89)	0.001ⱡ
Cohabiting (n = 28)	6 (17.65)	1 (5.56)	5 (31.25)	
Divorced (n = 38)	15(39.47)	10 (35.71)	5 (100)	
**Education**				
None (n = 68)	18 (26.47)	10 (18.37)	8 (46.67)	
Primary (n = 348)	91 (26.15)	53 (23.87)	38 (30.16)	
Secondary (n = 336)	72 (21.43)	45 (21.13)	27 (21.95)	
Tertiary (n = 63)	18 (28.57)	15 (37.50)	3 (13.04)	
**Household size**				
Small (≤3) (n = 340)	83 (24.41)	57 (25.90)	26 (21.67)	
Large (≥4) (n = 445)	113 (25.39)	63 (22.22)	50 (31.25)	
**Turnaround time for viral load (days) (Median, IQR)**	6: 3–7			
**Viral load suppression** * **(n = 201)**	190 (94.53)	119 (95.97)	71 (92.21)	
**Virologic failure (n = 201)**	11 (5.47)	5 (4.03)	6 (7.78)	

*define by a viral load of ≤1000copies/ml of blood ⱡ Chi Square for linear trend

### Use of viral load results for clinical decision making, and limiting factors

Approximately 89% of the participants with VL suppression were continued on same ART regimen while 5/11 of ART patients with virologic failure were continued on same first-line ART regimen. Five of the 11 participants with virologic failure were switched to a second-line ART regimen while one person had a substitution in the initial ART regimen. Details of these and other criteria for decision making are shown in Table 4.

**Table 4:** Use of viral load test results for clinical decision making among patients who initiated ART in 2013, 2014 and 2015 in three regional hospitals in Cameroon.

Characteristics	Virologic failure (n = 11)	Viral load suppression (n = 190)
**Decision category**		
Continuation of first-line, no substitution	5 (45.45)	170 (89.47)
Continuation of first-line, substitution	1 (9.10)	20 (10.54)
Switch of regimen	5 (45.45)	-
**Other criteria**		
Poor adherence	6 (54.55)	20 (10.54)
Adverse effects of ARVs	-	10 (5.26)
TB co-infection	-	1 (0.53)

## Discussion

The results of this study show that the current viral load testing uptake is very low in frequency and in the proportion of patients accessing a VL test at any given time during ART. The median turnaround time, from request of VL testing specimen to return of results, is 6 days. Viral load suppression is high in this study (94.5%) while only 5.5% of the patients had a virologic failure (VF). The proportion of treatment switch in the small number of patients with VF was inadequate. However, in the absence of clear national guidelines on VL monitoring during the time of the study, these findings should be interpreted with caution.

We learn from the study results that HIV infected individuals in these regions were beginning ART soon after diagnosis (IQR (time from diagnosis to start of ART): 1.5–5 months), which is a good thing. However, one would have expected a wider IQR because the immunologic criteria for ART enrolment have been in use during the time when most of the cohorts included in this study started ART. This finding has several implications. Firstly, some patients were initiated on ART at a high CD4 count. Secondly, it could be suggestive that some patients were often HIV-diagnosed clinically (contrary to voluntary testing) at low immunologic status and therefore enrolled into treatment and care earlier-this is possible considering the commitment of the government of Cameroon into campaigns against stigmatisation during the period from 2013 and beyond.

The overall and timely uptake of VL testing is very poor in this study. This was, however, improving with year of enrolment and duration on ART. It did not, however, fit into the recommendation of VL monitoring as none of the patients had done consecutive VL assessments for the duration on treatment. This finding of low access to VL testing is consistent with a global call for governments in resource-limited settings to commit politically and ensure scale up of viral load testing that align with the new guidelines for ART monitoring in the 21^st^ century. There are several possible explanations to the low VL testing uptake in these centres. An obvious explanation would be the lack of laboratory infrastructure for VL testing at the treatment centres. It is also possible as anecdotal reports suggest that laboratory equipment is available but the laboratory lacks human capacity and the reagents to conduct VL testing. As VL remains the key predictor of treatment success or failure in ART, low VL testing rates provide limited information about success in the ART programs. In Cameroon, VL testing is still centralised for most of the treatment centres involved in this study and many other treatment centres within the primary health care structure. To raise hopes towards achieving the United Nation’s AIDS (UNAIDS) program’s target of 90% viral load suppression in the population, political commitment and other leverages are required to strengthen large scale VL testing.

A turnaround time (TAT) of 6 days (IQR: 3-7days) is plausible when compared with reports from Malawi, where only the pre-analytic period of VL testing was found to be up to 39 days in 2015 [23]. The pre-analytic and post-analytic phases for VL testing are expected to be relatively longer for these treatment centres, since VL samples and test results require distant shipments. New VL testing platforms such as point of care tests (POC) will greatly improve access and shrink the TAT. This good turnaround time, however, disconnects enormously from the uptake of timely VL testing; for instance, approximately 65% of the patients did a VL test after being 24 months on ART. An obvious reason for the disconnect could be the absence of an operational plan for routine VL testing at the time of the study in conjunction with the fact that VL testing was usually adhoc, and could be offered at the discretion of the clinician. It is also possible that VL request were selectively offered for patients with the economic potential to pay. In the absence of universal access to VL testing in a country, the cost of VL test could be beyond the ability of several patients to afford in a timely manner even when offered.

The anticipated benefit of a VL test result lies on the readiness for a clinical decision to switch the treatment regimen or maintain similar regimen with enhanced adherence counselling. It is unfair to judge a clinical decision to switch or substitute a treatment regimen without considering information on adherence by the patient. It is possible that a patient who poorly adheres to the first line ART regimen would equally not adhere to the second line even if they are switched based on VF without enhanced adherence counselling. The criteria that were used for substitutions of first line regimens like features of drug toxicity, as in previous studies [24], and worsening clinical state, also affect patient adherence to ART regimens [25]. However, non-adherence from the available information in this study was almost as high as the frequency of switch to the second line regimen. It is therefore not surprising that only closed to half of the patients in our sample with VF (54.5%) were switched to the second line. This rate is lower than the rate of switching that has been reported from Uganda at 24 months (65.0%) following a VF [21]. A study has also reported an association between poor adherence and switch to second-line regimen [26]. In addition to the behaviour of patients while receiving a specific regimen, the ability of care providers to take a clinical decision to switch also depends on the availability of second-line and even third-line ARV regimens.

The ministry of public health in Cameroon is presently making efforts with the support of many global health partnerships to ensure improved access to VL testing across all regions of Cameroon. The available conventional laboratories underway for full functionality will greatly support the achievement of the third ‘90’of the UNAIDS targets [1] in Cameroon. Strategic planning for implementation of VL cannot be overstated now when we want to push for an end to AIDS.

This is the first study from Cameroon that demonstrates the uptake and utilization of viral load test results for HIV patient management at a time when VL testing is the preferred laboratory monitoring strategy. The strength of this study lies in its large sample size, multi-site nature which to an extent attempts to paint a national picture about VL testing in Cameroon. We were limited in our findings of low VL testing rate which made it inappropriate to infer the information about switch of regimen, since only a small number of patients were failing treatment. This study did not explore the availability of second-line ARVs, which could give more clarity on factors limiting the switch to second-line regimen. We cannot also account for the quality of the data collected as the study design dictates. However, the large sample size improves the precision of our findings.

## Conclusion

The uptake of viral load monitoring of ART in North West, South West and West Regions of Cameroon is inadequate. The turnaround time for VL testing is plausible. Adequacy of switch to second line regimen is low, but can be best exploited with larger number of patients testing for VL. Political action and other leverages is needed to strengthen the scale up of VL testing in Cameroon.

## Supporting information

S1 Dataset Viral load testing in three regions of Cameroon. (ZIP)
